# Shedding Light on the Main Characteristics and Perspectives of Romanian Medicinal Oxygen Market

**DOI:** 10.3390/healthcare9020155

**Published:** 2021-02-03

**Authors:** Adriana AnaMaria Davidescu, Simona Andreea Apostu, Cristina Stanciu-Mandruleanu

**Affiliations:** 1 Department of Statistics and Econometrics, Bucharest University of Economic Studies, Bucharest 010374, Romania; simona.apostu@csie.ase.ro; 2 Labour Market Policies Department, National Scientific Research Institute for Labour and Social Protection, Bucharest 061643, Romania; 3 Romania Institute of National Economy, Romanian Academy House, Bucharest 050711, Romania; 4 Economic Cybernetics and Statistics Doctoral School, Bucharest University of Economic Studies, Bucharest 010374, Romania; cristina.mandruleanu@rdsmail.ro

**Keywords:** medicinal oxygen, market perspectives, Romania, statistical survey, O93 oxygen, multilevel approach

## Abstract

Medicinal oxygen plays an important role in healthcare, being essential for the existence and maintenance of the health of millions of people, who depend on medicinal oxygen every day, both in hospitals and at home. Medicinal oxygen is the primary treatment administrated to the majority of patients suffering from respiratory problems and low levels of oxygen in the blood, and in the context of the actual health crisis caused by the new COVID-19, the challenge is represented by increasing the supply of medicinal oxygen while reducing cost so that it is accessible where it is needed most, free at the point of use. It will take increased investment and commitment to put oxygen at the center of strategies for universal health coverage. In this context, it becomes essential to investigate the main characteristics of the Romanian market of medicinal oxygen, highlighting top key players, market development, key driving factors, types of products, market perspectives as well as shedding light on the segmentation of this particular market based on considerations regarding regions, hospital competence class and hospital specialization. Also, the research aims to explore the regional disparities in the decision of using O93%medicinal oxygen, revealing the main factors related to the usage of this type of product among Romanian public hospitals. The research relies on the first quantitative survey regarding medicinal oxygen usage among 121 public hospital units from a total of 461 public hospitals in 2018, which meet the specific requirements: includes the entire population according to the list published on the website of the Ministry of Health, is the most recent data and does not show repetition. The sampling was of probabilistic stage-type stratification, with the following sampling layers: hospital county distribution, hospital competence class officially assigned by the Ministry of Health and also area of residence (urban/rural). In order to analyze the main characteristics of the Romanian oxygen market, the following methods have been used: analysis of variance (ANOVA) together with Kruskal–Wallis, Pearson correlation coefficient as well as Goodman and Kruskal gamma, Kendall’s tau-b and Cramer’s V, as well as multilevel logistic regression analysis using hierarchical data (hospitals grouped in regions). The Romanian market of medicinal oxygen is rather an oligopoly market characterized by the existence of a small number of producers and two types of products currently used for the same medical purpose and having a substitutable character: medicinal oxygen O99.5%, and medicinal oxygen O93%. An overwhelming proportion of public hospitals agree that both types of medicinal oxygen serve the same therapeutic purpose. The Romanian market of medicinal oxygen highlighted a significant segmentation on considerations based on regions, hospital competence class and hospital specialization. Regarding the main perspectives, the Romanian market of medical oxygen keeps the growth trend registered globally, with development perspectives for competitors. Exploring the regional disparities in the decision of using O93 medicinal oxygen, the empirical results acknowledged the important role of unitary price, hospital capacity and the relevance of this product seen as a medicine. Medicinal oxygen is vital in sustaining life, proving its utility mainly in the context of the actual health crisis. In this context, the Romanian local market exhibits prospects for further development, being characterized by an important segmentation depending on regions, hospital competence class and hospital specialization.

## 1. Introduction

Medicinal oxygen plays an important role in healthcare, being essential for the existence and maintenance of the health of millions of people who depend on medicinal oxygen every day, both in hospitals and at home. Respiratory diseases registered an ascending trend, and by 2030, the World Health Organization predicts that they will become the third leading cause of death globally. Oxygen plays an essential role in care, being used in mechanical ventilation both for patients with chronic obstructive pulmonary disease and severe asthma, and for those suffering from a wide range of respiratory conditions, in operations requiring general anesthetic, in neonatal wards as a respiratory support, etc. In addition to therapeutic applications, oxygen is used in calibration gas mixtures and to verify and maintain the reliability and accuracy of various medical devices and diagnostic equipment or as an oxidizing agent and catalyst in various scientific and industrial processes.

In the actual context of the health crisis caused by the new coronavirus (COVID-19), we expect the demand for medicinal oxygen to increase even more. The World Health Organization recommendations suggest the development of medicinal oxygen systems and provision of pulseoximeters to measure blood oxygen levels in all countries, with the challenge being represented by increasing the supply of medicinal oxygen while reducing cost so that it is accessible where it is needed most.

The main purpose of this study was to provide a first exploration of the Romanian market of medicinal oxygen, collecting data on the total consumption of medicinal oxygen by Romanian hospitals, as well as the total value of hospital expenditures for medicinal oxygen supply. It is worth mentioning that for the Romanian oxygen market, there are currently no relevant data, articles or evolution estimates. The paper addresses in particular an insufficient subject treated in the medical and economic literature, namely, the medicinal oxygen produced by oxygen concentrators (O93%) and that produced industrially in factories and plants (O99%). 

The lack of a bibliography dedicated to this subject is mainly due to the fact that medicinal O93 oxygen is the only drug in the world produced by a medical device (oxygen concentrator) that is in accordance with the European Medical Devices Directive 93/42/EEC, produced by a new technology compared to the classic one. National and European health authorities did not have appropriate rules and legislation in place to allow the widespread use of O93 oxygen in all health facilities, due to the novelty of the production technology that appeared in the 1980s although it was regulated as a drug in the American Pharmacopoeia for the US market.

Within the study, a special attention has been grounded on the identification of the therapeutic equivalence of the two types of medicinal oxygen (O93% and O99%), presenting the main actions undertaken by one of the market leaders, Microcomputer SA. The validation of the medical therapeutic equivalence is based on both a technical memorandum issued by experts in the field and the 20 years of medical practice in over 100 hospitals in Romania, in which medicinal oxygen O93% was used in all medical procedures without any qualitative difference from O99%. Obtaining and analyzing this information is essential for new companies interested in entering this market, to increase their chances of rapid integration. The research carried out in this study aims to provide a relevant image of the real state of the market, of the economic potential, of the expansion potential and of the position that the economic agents have on the market, pointing out the following aspects: the current market size and evolution, key market players and market shares held by them, the main characteristics of sanitary units and the main barriers encountered by a new player in the market.

It is important for any competitor to highlight its ability to maintain and expand the market relative to the capabilities of other competitors, to be able to build predictions for performance and risks and to be able to estimate the direct and indirect effects of launching a new product or equipment. The company, before deciding on a strategy, must understand and analyze the market it is in in terms of structure, dynamics and possible developments. The purpose of this research is to achieve a very important analysis tool for the study of strategies to be promoted. Thus, the paper aims to answer to the following main question: “What are the main characteristics and perspectives of the Romanian market of medicinal oxygen?” Finding a reliable answer to this question will shed light on a niche market, unexplored so far, also providing solutions that will help the main actors from the market in planning their development strategies, mainly in the context of an actual health crisis.

The article brings its contribution to the literature in the following four ways. Firstly, this research is, to our knowledge, the first empirical study regarding the medicinal oxygen market in Romania based on survey data collection, with the information obtained offering valuable insights into the main actors from the market to establish: the share held in the total market and product market, the geographical area covered by own sales, number and categories of consumers to whom the product is addressed and the specific purchasing power of the target consumers.

Secondly, this study represents the first market prospecting, highlighting the market profile, the context in which the activity takes place, the institutions that regulate it, the main actors, the types of products sold, the development trends, as well as, among others, the factors underlying the expansion. At the level of Romania, the characteristics of an oligopoly can be identified, which means the product market is formed by a small number of companies.

Thirdly, the research refers to the first quantitative analysis of medicinal oxygen market perspectives, with major implications in substantiating future expansion decisions.

Fourthly, the paper offers valuable information regarding the adoption of the first medical and legal European decision for therapeutic equality treatment of two types of products, O93% and O99% medicinal oxygen, as well as presenting proposals concerning the improvement of the standard SR EN ISO (Romanian Standard European Standard International Organization for Standardization) 7396-1: 2016, related to the equal treatment between O93% and O99%, as well as proposals for revision of Document 195 issued by the European Industrial Gas Association, Belgium (EIGA).

All these regulations of the Romanian medicinal oxygen market have been detailed in the Supplementary Material.

The paper is structured as follows. Section 2, materials, offers an overview of the most relevant studies regarding the topics of medicinal oxygen and Section 3 outlines a theoretical background of the methods used in the quantitative analysis. Section 4 details the empirical results. The last section presents discussions and the main conclusions of the research.

## 2. Materials

Oxygen is an essential medical therapy that has saved lives for over 100 years [1], being include by the World Health Organization (WHO) on the list of essential drugs [2]. Medicinal oxygen has multiple indications, being a drug without an alternative agent, with the WHO indicating its importance in the care of seriously ill children [3], emergency services, anesthesia and surgery, both in national and provincial hospitals [4].

Oxygen is not available in all primary care units and is often lacking in national hospitals [5], and in hospitals it has been reported to be expensive for patients [6]. As the overall use of oxygen has increased, it has been necessary to develop the oxygen home care industry, with Yong et al. [7] indicating that the new oxygen strategy at home has improved the health of patients with COPD (chronic obstructive pulmonary disease). 

Changes in the healthcare market are due to both therapeutic developments and new care technologies [8]. The medical industry attaches great importance to services due to the impact they can have on customers [9]. Medical equipment has developed business strategies, providing patient-centered care, design thinking and designing services. Lee [10] studied the design of outpatient care from the perspective of service design, focusing on healthcare, indicating that the characteristics of service delivery are classified into factors of environmental conditions and functionality, being correlated with satisfaction, facility, perceived quality of care and approach behavior.

Therapeutic oxygen is provided to patients, representing an important and growing segment of the medical industry, being provided in both liquid and compressed form [11].

Oxygen represents a life-saving therapy and should be prescribed accurately with the required flow rate and delivery device [12], with it being scientifically proven that both 93% oxygen and 99% oxygen provide the same quality of patient care [13]. 

In medicine, oxygen has a therapeutic role in order to prevent or correct arterial hypoxemia and any resulting tissue hypoxia, a serious and common disease, in urgent cases involving trauma, respiratory distress and circulatory disorders [14,15]. For a patient with chronic obstructive pulmonary disease who has tissue hypoxia from acute hypoxemia of pulmonary origin, oxygen treatment has a beneficial effect, both in the short and long term [16,17].

Regarding the process of obtaining medicinal oxygen, globally, there are two ways: oxygen concentration 99.5% obtained industrially, by fractional distillation of liquid air by the cryogenic method. Oxygen is supplied in this case either through liquid oxygen tanks or compressed oxygen tanks, and oxygen concentration 93% ± 3%, produced by separating the air by the method of molecular sieves (PSA (Pressure swing adsorption) method). This type of oxygen is produced on the spot by means of a medical device, computerized technology that appeared after 1990.

In Romania, there is no information about the medicinal oxygen market or analyses regarding the market characteristics, evolution trends, type of competition or competitors. Thus, this paper aims to obtain information and process data on the Romanian oxygen market, in order to define the medicinal oxygen market: product market, company market, competitive market of the company studied on the total market, market dynamics and characteristics.

Romania is the only country in the European Union where the Industrial Gas Association has complained that O93 oxygen is not a medicine, is not therapeutically equivalent to industrially oxygen and VAT should not be applied to the 9% medicine. Following the fact that the company that introduced this medicinal oxygen on the Romanian market for the first time was claimed in court, a trial in court took place during 5 years, finalized at the High Court of Justice and Cassation with a final sentence in which established the following:

- 93% medicinal oxygen has similar therapeutic effects to 99.5% oxygen because the active substance is the same in both products in accordance with European Directive 2001/83/EC;
- 93% medicinal oxygen does not require a marketing authorization because, on the one hand, for oxygen it is sufficient to authorize the medical device, i.e. the oxygen concentrator, the oxygen being obtained by a non-industrial process, and therefore does not require authorization, and on the other hand, oxygen is continuously monitored by an oxygen analyzer, which permanently displays the concentration, and if it does not correspond it is automatically switched to the reserve oxygen source (which is not happens with industrially oxygen, thus requiring a marketing authorization);
- 93% oxygen is a drug according to the article 695 of Law 95/2006.

Medicinal oxygen is administrated as additional oxygen, normally contained in ambient air, to patients with respiratory diseases, but not only this as it is also used for a variety of respiratory ailments, from chronic obstructive pulmonary disease to cystic fibrosis to sleep-related breathing disorders, from acute treatment to long-term therapy [18]. The area of oxygen application is supported by the versatility of its therapy products, which continue to become more lightweight, more portable and more cost-effective for patients and insurers [19]. 

Although most studies pointed out the benefits of oxygen therapy, when used non-specifically for medical emergencies, it can have negative effects on the body [20,21], and in the case of hyperbaric oxygen administration [22,23], Siemieniuk et al. [24] highlighted that too much oxygen increases mortality for hospital patients, so it must be administered with great care. 

A target SpO_2_ range of 90–94% is effective for most patients and 88–92% for patients at risk of hypercapnic respiratory failure [24]. O_2_ is used to treat patients with respiratory problems, which is one of the main causes of death, with approximately 5% of the world’s population suffering from these diseases [25,26]. 

The medicinal oxygen concentrator (MOC) usually produces oxygen of 90–93% concentration, an O_2_-enriched gas from the surrounding air, at a rate of ≤10 L/min (LPM) for individual use [27]. Oxygen concentrators have been used in modern countries to provide medicinal oxygen for home medical services, proving to be very reliable [28]; therefore, a novel design of a compact rapid pressure swing adsorption system is proposed for application as a medical oxygen concentrator [29]. 

Oxygen 93% is a relatively new product, made by oxygen concentrators developed by the medical technology market, and it is used by patients with high oxygen needs, based on the use of electricity [30]. O93% is a gas produced by a molecular sieve process, containing more than 90% and less than 96%, the rest being argon and nitrogen, as defined in USP (United States Pharmacopoeia (USP)) in 1984 [31]. Depending on the limitations of the molecular sieve technology of the time, Friesen pointed out that for a primary oxygen supply, it is necessary for each hospital to adopt O93% oxygen [32]. 

The legal working document of the European Directorate for the Quality of Medicines and Health Care (EDQM, body of the Council of Europe) was the basis for the introduction of the O93% monograph in the European Pharmacopoeia in March 2010 in Strasbourg (European Directorate for the Quality of Medicines and HealthCare (EDQM) of the Council of Europe). Considering that the monographs of the pharmacopoeia represent an official standard, the regulation regarding the quality of oxygen medicine was implemented directly in the Romanian national legislation by the Health Law 95/2006, republished regarding the health reform. 

In the process of transferring the oxygen monograph from the American Pharmacopoeia to the European Pharmacopoeia, have been presented the medical results and conclusions of the use of medicinal oxygen O93% in over 100 hospitals of Microcomputer Service company, the leader of the market, that used in Romania the oxygen produced by generators since 2000.

Each type of oxygen corresponds to a monograph in the Pharmacopoeia, as follows: the medicinal oxygen produced by O93% generators is found in monograph 2455, and the liquefied medicinal oxygen, industrial product in monograph 0417.

Both monographs state that the two types of oxygen are intended for medical use, which implies that they are similar products and have the same purpose. The medical quality of medicinal oxygen O93% has been clearly legislated by the only reference work for the control of drug quality in the European Union, becoming mandatory for all signatory states of the convention. Since the introduction of the O93% medicinal oxygen monograph in the European Pharmacopoeia, healthcare units have gradually introduced the need to comply with public procurement documentation according to the oxygen provisions.

The regulation regarding the quality of medicinal oxygen was implemented directly in the national legislation by the Health Law 95/2006 regarding the health reform [33]. 

The evidence indicates that medical devices such as oxygen concentrators are feasible and cost-effective for the administration of oxygen therapy, especially if oxygen tanks and piped oxygen systems are inadequate or unavailable. The good quality of oxygen concentrators makes them appropriate as a sustainable and safe source of oxygen for multiple patients. 

The usefulness and effectiveness of oxygen concentrators have been proven by increasing access to oxygen and improving the overall quality of care in low-resource countries. Studies in Egypt, Gambia, Malawi, Nepal, Nigeria and Papua New Guinea highlighted the use of concentrators in order to expand oxygen availability in health facilities in resource-limited countries [34]. These studies indicated that oxygen concentrators have been used successfully in developing countries to provide oxygen to children and adolescents in surgery.

The advantages of oxygen concentrators have been discussed in the technical literature, and these include high reliability and low costs compared to oxygen cylinders and oxygen supply pipelines. Disadvantages of oxygen concentrators include the need for routine maintenance and a reliable power source, with both being addressed through effective planning and preparation. Collaboration between management, clinicians and technicians is needed to ensure the efficient implementation and timely maintenance of concentrators [35]. 

Various consulting firms and research have provided valuable information regarding the main characteristics of the global market of medicinal oxygen through different research reports (Market Watch, Business Wire—2016, Top Tribune—2017, Industrial Oxygen Report, Future Market Insights, British Medical Research Council, North American Nocturnal Oxygen Therapy).

An important document acknowledging the role of the global oxygen market and its future perspectives is the Industrial Oxygen Report published by the Global Marketers Research Center [36], providing valuable information about the oxygen industry, objectives, market definition, the field of industrial oxygen and market size estimation, analyzing the growth factors, industrial plans, policies and development strategies implemented by the main industrial oxygen actors. The internal market of home oxygen therapy was analyzed by Garattini et al. [37] in five European countries (Denmark, France, Germany, Italy and the United Kingdom) according to domestic legislation, prescription procedures, delivery and market, with the results indicating no specific relationship between the health system and the oxygen therapy market, and home care being important within the national health services. 

Regulation of the medicinal oxygen regime 93% ± 3% represents an essential step in the future development of the market for medicinal oxygen produced by concentrators, taking into account the economic and financial power of direct competitors.

Efforts to improve the legal framework and develop alternatives to oxygen supply hospitals continued beyond the competence of local authorities and the company argued to recognized national and international standardization bodies for clarification on this issue.

In the context of the health crisis caused by COVID-19, the demand for oxygen increased. This increase is attributed to the supply of oxygen, which plays a significant role for patients suffering from chronic disorders in order to maintain the required level of oxygen, this factor is expected to fuel market growth [38]. Therefore, the World Health Organization recommends the development of medicinal oxygen systems and provision of pulse oximeters to measure blood oxygen levels in all countries. According to the Every Breath Counts Coalition [39], the COVID-19 response so far has largely overlooked the importance of medicinal oxygen supply and diagnostic tools for identifying hypoxemia. 

It will take increased investment and commitment to put oxygen at the center of strategies for universal health coverage [40] in order to increase the supply of medicinal oxygen while reducing cost so that it is accessible where it is needed most, free at the point of use. 

The availability of medicinal oxygen was analyzed by Nabwire et al. [41] in pediatric wards in 11 hospitals in eastern Uganda. The results indicated that oxygen delivery was available in 18% of hospitals and of the six concentrators found, two did not work at all and two produced a flow of O2 < 80% pure [42]. Oxygen therapy is very important in order to save lives, reducing mortality caused by pneumonia by about 35% [43], therefore it should be available in all hospitals. The same results regarding oxygen therapy availability were highlighted especially in the case of children’s hospitals [44,45]. 

Most hospitals face smaller amounts comparing to the need for oxygen [46], but delivery problems and its use have also been identified. The most common situations reflect the availability of equipment required for oxygen delivery and unavailability of oxygen concentrators [47]. Studies in different countries on medicinal oxygen indicate that access to oxygen equipment, clinical guidelines, training and technical support are missing [48,49,50,51,52].

Another important criteria considered in the case of oxygen therapy is represented by cost, with the oxygen price influencing the oxygen delivery in small hospitals in developing countries [32].

Cost influences overall health, followed by wasteful time and resources [53,54,55,56,57,58], with healthcare expenditure significantly influencing the individual’s health status [59].

Regarding healthcare, changes in supply, access and funding have been highlighted [60], and from the perspective of health financing, the most important is the compulsory health insurance for all citizens [59]. Although citizens benefit from compulsory insurance, the introduction of taxes for using public and private facilities has led to an increase in the costs supported by the population [61], and some patients risking falling into ‘the medical poverty trap’ [62]. Therefore, it is necessary to rationalize spending so as to ensure wider and better access to medicines [63], mainly for low-income countries.

Considering that the poor population have few financing options, with the delivery and financing of health services being affected [63], ‘radical health sector reforms’ are needed in order to supply expensive drugs and sophisticated technologies [64].

For poor countries, private health services used by patients require very large amounts and more often in the treatment periods, incomes were interrupted, with drug taxes and costs being exceeded [65]. Therefore, poor people are most exposed to risk of destitution following market-based healthcare reforms [66].

Depending on the method of financing, the health systems used in Europe are the Bismarck system, the Beveridge-type national health system, the Semasko-type centralized health insurance system and the private health insurance system. In Romania, since 1999, a Bismarck type health system has emerged, but it also has influences from the Semasko and Beveridge models and the Social Health Insurance Law has been implemented. The characteristic of the structure of this system is that the financing is realized through the contribution for health, which is obligatory both from the employer and the employee [67].

According to the annual report “Health in the European Union” [68], Romania allocates to the health system the least money in the European Union, both per capita and as a percentage of Gross Domestic Product, the result being an inefficient health system, unable to save lives. Almost half of the deaths registered in Romanian hospitals could have been avoided given the technological evolution and current medical knowledge, according to data provided by Eurostat. In this context, the poor population face the risk of not being able to afford private services or treatment, facing medical poverty.

Having a two-fold objective, on the one hand to investigate the main characteristics of the Romanian market of medicinal oxygen and on the other to analyze the main characteristics of a particular type of product (the O93% medicinal oxygen), exploring the main drivers in adopting the decision of using this type of product, the following research questions were asked to seek the answer in this research: 

What are the main actors on the medicinal oxygen market? What are the main characteristics of the Romanian market of medicinal oxygen? What are the main methods of supplying the medicinal oxygen? What are the main types of products used on the market? What are the market perspectives? What is the level of price and consumption on this market? Is there a segmentation of medicinal oxygen market based on regions, hospital competence class and hospital specialization considerations? Is O93% medicinal oxygen frequently used among Romanian hospitals? What influences the decision of using O93% medicinal oxygen? 

The paper fills the gap in the literature by exploring a subject treated insufficiently in the medical and economic literature, the medicinal oxygen produced by oxygen concentrators (O93%) and that produced industrially in factories and plants (O99%). 

The lack of a bibliography dedicated to this subject is mainly due to the fact that medicinal O93% oxygen is the only drug in the world produced by a medical device (oxygen concentrator). 

**Figure 1:**
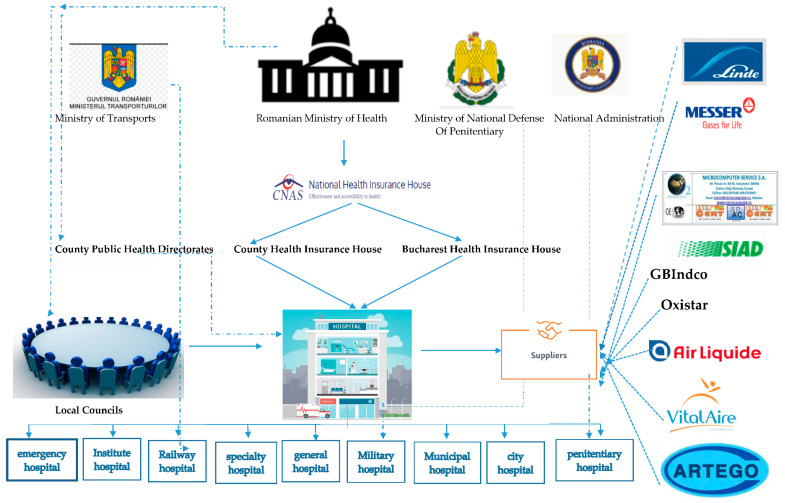
Main actors of the Romanian oxygen market.

The Romanian market for medicinal oxygen is characterized by the existence of a small number of producers and two types of medicinal oxygen currently used in the Romanian hospital system: medicinal oxygen O99.5%, produced according to liquefaction technology, being obtained industrially and delivered to tubes and tanks, and medicinal oxygen O93%, being produced according to the technology of molecular sieves and using medicinal oxygen concentrating devices, which allows the preparation of medicinal oxygen right inside the hospitals. The main actors of the Romanian oxygen market are presented in Figure 1.

Based on the research questions, the following hypotheses have been formalized in order to highlight the main characteristics of the Romanian medicinal oxygen market:

**Hypothesis** **1** **(H1).** *There are regional disparities regarding the overall medicinal oxygen market (consumption, price, type of products (oxygen O93%, oxygen O99%)). The need to evaluate this regional segmentation component is based on the identification of regional/county poles, in order to establish the regions that could attract the interest of suppliers regarding the hospital profitability in the area, in terms of medical oxygen acquisition. The regional consumption segmentation could be justified by the hospitals’ competence class and specialization.*

**Hypothesis** **2** **(H2).** *There are significant differences regarding the overall oxygen market (consumption, price, and type of products (oxygen O93%, oxygen O99%)) based on hospital capacity, competence class and hospital specialization and suppling method. The main motivation for this type of segmentation is that it allows the division of the target market into groups and segments according to these criteria, thus facilitating knowledge of market characteristics that a potential company would like to operate. By collecting all the results, the oxygen supplier understands the problems it solves by offering products or services, profiles hospital units, current or potential customers and segments target customers, thus determining the type of products/services offered and the corresponding price.*

**Hypothesis** **3** **(H3).** *Main suppliers divide the oxygen market using different supplying methods and supplying hospitals with different specializations.*

*The main suppliers of the medicinal oxygen market are the suppliers of both medicinal oxygen produced by oxygen concentrators/generators on site, and liquid oxygen manufactured industrially and delivered through tanks in storage tanks installed in sanitary units. The delivery price of the medicinal oxygen to hospitals plays an important role, because oxygen purchases are made from public money, through a national public procurement platform, in order to determine the most advantageous offer in terms of economically according to the provisions of the public procurement law.*

**Hypothesis** **4** **(H4).** *There are significant differences in supplying method based on hospital competence class and hospital specialization.*

*Depending on the hospital competence and specialization, it addresses different diseases and treatments, therefore the demand for medicinal oxygen and the supply method are different.*

**Hypothesis** **5** **(H5).** *The decision of using O93% medicinal oxygen relies primarily on price and hospital capacity and secondly on hospital specialization and on the future perspectives of the medicinal oxygen market.*

From our point of view, this hypothesis is even more important, because according to the law on public procurement, hospitals must establish the most economically advantageous offer based on the award criteria and evaluation factors provided in the procurement documents. In this sense, the following award criteria can be applied: the lowest price, the lowest cost, the best quality–price ratio and the best quality–cost ratio.

The decision to use medicinal oxygen O93% is based primarily on the financial component, the purchase price of medicinal oxygen, also taking into account other factors, namely: product quality, technical advantages, functional characteristics, accessibility, design concept, ease in use, the qualification of the personnel designated to handle the product, after-sales services, technical assistance, delivery conditions and warranty period.

It could be relevant to identify what are the factors on which this decision of using O93 medicinal oxygen are based on, taking into account that the main advantages of using O93 medicinal oxygen compared to industrially produced oxygen (O99%) are:

- Decreasing the costs of the hospital for the supply of medicinal oxygen compared to the old method, by 15–20%.
- The execution time of the project will be very short, respectively the installation period of the installation will be a maximum of 2 weeks.
- Working at pressures close to the supply of the devices, 2–7 bar, eliminating the danger of working with containers loaded at very high pressures (150 bar).
- Providing medicinal oxygen in a permanent regime, at low pressures that are not dangerous for the human body.
- Elimination of human effort to handle purchased oxygen tubes from oxygen production stations used for industrial purposes.
- Elimination of the effort to procure oxygen tubes in a timely and safe manner, as a concern of the hospital.
- Elimination of the transport that in the winter period constitutes an impediment in the provision in a constant rhythm with oxygen according to the requirements of the hospital.

## 3. Statistical Method

The research relied on a quantitative survey conducted based on a structured questionnaire among 121 sanitary units using all Romanian hospitals, which meet the specific requirements, as a survey base: includes the entire population according to the list published on the website of the Ministry of Health, and is the most recent data and does not show repetition. The questionnaire based on 12 items was sent by email to 121 public hospital units from a statistical population of 461 public hospitals.

The sampling was of probabilistic stage-type stratified. The sampling layers targeted were hospital county distribution, hospital competence class and area of residence (urban/rural). The data collection has been undertaken during the period March–July 2018. Considering the sample size, we do not face the high risk that observations are due to chance, with tiny and small associations also being detected.

The questionnaire was sent by email to the hospital units. The questions were formulated precisely, without ambiguity, so as to ensure that the necessary data were obtained to meet the research objectives.

The first part of the questionnaire aimed to find out the information related to the potential of each consumer related to a certain period of time, respectively the average and monthly value consumption of the registered medicinal oxygen. The following questions concerned information on the type of product used by hospitals, its characteristics and the level of knowledge of it by consumers. Also, answers were requested indicating some characteristics of the hospitals, in order to classify them later.

During the survey, the community of hospital units has been dived into classes as homogeneous as possible, in order to have similar characteristics. The division of hospitals into classes was carried out according to the competence class officially assigned to hospitals by the Ministry of Health, as follows:

- Hospitals with very high competence, which corresponds to the structure of the regional emergency hospital.
- Hospitals with high competence, including the hospitals that serve a county and/or neighboring counties, providing medical services of great complexity.
- Hospitals with average competence refers to hospital units that serve their own county and, in particular, neighboring counties. This category includes non-clinical county emergency hospitals, excluding units from university centers.
- Hospitals with basic competence, referring to hospitals that serve the administrative-territorial unit, being represented by medical service units with a low degree of difficulty.
- Hospitals with limited competence, respectively hospitals that provide medical services for hospitalization for chronic diseases or in a single specialty.

The units were extracted by the random number table procedure. The sample is representative of the hospitals’ population with an error of ±4.9% at the 95% confidence level. The data was collected during the period March–July 2018.

The questionnaire contains information regarding the average monthly oxygen consumption recorded by hospitals in 2017 (m^3^/month), the average monthly amount spent by the hospital for the supply of medicinal oxygen in 2016 as well as the main way of suppling medicinal oxygen (Table 1).

The second section of the questionnaire was related to the type of product used by hospitals, its characteristics and its level of knowledge by consumers as well as the main suppliers on the market.

The questionnaire ended with future changes in the oxygen consumption, taking into account a targeted future expansion of the hospital and how this expansion will affect the overall level of consumption. 

Within the survey, additional information related to hospitals has also been collected regarding the competence class, the type of specialization, number of beds and number of operating rooms (Table A1).

In order to analyze the oxygen market, descriptive frequency tables and statistics (mean and standard deviation) have been used, as well as pie charts and bar graphs. The analysis of variance (ANOVA) together with the Kruskal–Wallis test have been used to highlight the main differences regarding the medicinal oxygen market characteristics by regions, hospital competence class and specialization. Also, the Pearson correlation coefficient as well as Goodman and Kruskal gamma, Kendall’s tau-b and Cramer’s V have been used to highlight relevant and statistically significant associations between the main characteristics of the oxygen market. The Statistical Package for Social Sciences version 20 (SPSS, IBM Corp, Armonk, NY, USA) was used to perform the analysis.

In order to explore the regional disparities regarding the prevalence of using O93% medicinal oxygen, multilevel logistic regression analysis using hierarchical data (hospitals grouped in regions), reflecting group variability and group-level characteristics’ effects on outcomes at the individual level, has been applied. STATA (StataCorp, LLC, Lakeway Drive, College Station, TX, USA) software 15 was used to perform the analysis.

In order to analyze the between-region variation in the prevalence of using O93% medicinal oxygen, several two-level models were estimated. 

Firstly, the appropriateness of the multi-level approach was tested by the estimation of a baseline random intercept model without any explanatory variables, the empty two-level model with only an intercept and country effects (the null model) has the following specification:

(1) $$\log\left(\frac{\pi_{\mathrm{ij}}}{1-\pi_{\mathrm{ij}}}\right)=\beta_{0}+u_{0j}$$

The intercept $\beta_{0}$ is shared by all countries, while the random effect $u_{0j}$ is specific to county j and it follows a normal distribution with variance $\sigma_{u0}^{2}$. 

Secondly, a first-level (i.e., individual level) characteristics model was estimated in an attempt to understand their effects:

(2) $$\log\left(\frac{\pi_{\mathrm{ij}}}{1-\pi_{\mathrm{ij}}}\right)=\beta_{0}+\beta_{1}\cdot X_{\mathrm{ij}}+u_{j}$$

In the random intercept models, the model intercept varies randomly across regions and the main assumption was that the coefficients of all explanatory variables are fixed across regions. 

Thus, assuming that the decision of using O93 medicinal oxygen could vary across regions depending on price level and the hospital capacity (total number of beds), random slope models have been estimated, allowing for both the intercept and the coefficient of these two explanatory variables to vary randomly across regions. 

In a random slope model, a group-level random term u_j_ has been included as a linear predictor of the model.

(3) $$\log\left(\frac{\pi_{\mathrm{ij}}}{1-\pi_{\mathrm{ij}}}\right)=\beta_{0}+\beta_{1}\cdot X_{\mathrm{ij}}+\beta_{3}\cdot X_{\mathrm{ij}}\cdot u_{1j}+u_{0j}$$

${\mathrm{where}\;X}_{\mathrm{ij}}\cdot u_{1j}$ is a new term to the model, 0 is the subscript for the intercept residual and random effects $u_{1j}{\;\mathrm{and}\;u}_{0j}$ are normally distributed with the variances $\sigma_{u1}^{2}\;\mathrm{and}$ $\sigma_{u0}^{2}$ and the covariance $\sigma_{u01}^{2}$.

The extension from random intercepts to random slopes has introduced two new parameters to the model—$\sigma_{u1}^{2}$ and $\sigma_{u01}^{2}$—carrying out a test of the null hypothesis that both are equal to zero.

Regions showing an above-average positive relationship between hospital capacity and prevalence of using O93% medicinal oxygen will have $u_{1j}>0,$ while regions with a below-average positive (or possibly negative) relationship between hospital capacity and prevalence of using O93% medicinal oxygen will have $u_{1j}<0$.

In order to test whether the effect of unitary price and hospital capacity varies across regions, a likelihood ratio test was applied, taking into account the difference in the log likelihood values between the model with and without the random slope on these two variables.

## 4. Procedure

### 4.1. The Romanian Hospital Profile

Drawing the profile of the Romanian hospitals, we can mention the following:

- Most of them are located in the South-East region (19%), followed by the Bucharest-Ilfov region (17.4%).
- Most of the institute and general hospitals were located in Bucharest and in large counties, with a population between 500,001 and 750,000 inhabitants, while emergency hospitals were mostly distributed in counties with a population between 500,001 and 750,000 inhabitants (35.3%) as well as over 750,000 inhabitants (41.2%), and railways, military, municipal and specialty hospitals were mostly located in counties with a population between 500,000 and 750,000 inhabitants.
- The Romanian hospitals are not very well equipped, having 339 beds on average and 6 operating rooms (Table 2).
- Most of the Romanian hospitals (36.4%) provide medical services in a single specialty (infectious diseases, recovery, chronic, psychiatry), while 15.7% of them are municipal hospitals, which usually serve the county population in the administrative-territorial area. Only 14% of them are emergency hospitals that have of a complex structure of specialties, 13.2% are city hospitals, 5% institutes, 5% CF (railways) hospitals, 5% general hospitals, 4.1% military hospitals and 1.7% penitentiary hospitals.
- Of the hospital units, 36% have basic competence and only 7% of them have very high competence: 14% of them have high competence, 32% limited competence and 11% medium competence. Most of the hospitals with very high and high competence, as expected, were situated in the most developed region Bucharest-Ilfov, while most of the hospitals with rather limited competence were situated in the South-East region.

The empirical results of the Kruskal–Wallis test (Chi-Square = 27.75, *p*-value = 0.00) revealed that the North-East region was considered to be the region with the highest level of hospitals’ average competence, and only South-Muntenia region was placed at the opposite side, with a rather limited average hospitals’ competence.

### 4.2. Analyzing the Regional Segmentation of the Romanian Medicinal Oxygen Market

Reporting the annual consumption of medicinal oxygen to the total number of beds in the hospital, proxy for the hospital full capacity, we are able to build a performance indicator related to the medicinal oxygen consumption and to investigate its relationship with the unitary price, together with some significant differences among regions, competence class or specialization of Romanian hospitals.

Thus, the average value of medicinal oxygen consumption in the sample was 146.02 m^3^, with most of the hospitals registering a value of 120 m^3^ and only 25% of the hospitals included in the analysis reported a consumption higher than 205 m^3^. Related to the unitary price, it can highlighted that the average value for the entire sample of hospitals was 2.159 RON/m^3^, and only 25% of the hospitals paid more than 2.75 RON/m^3^ (Table 3).

The empirical results of the ANOVA revealed the existence of regional statistically significant differences regarding both the annual consumption per bed and also the unitary price (RON/m^3^), at the 10% level of significance, revealing that Bucharest-Ilfov and North-East were the regions with the highest level of average consumption reported to the total number of beds, while at the opposite side, there was South-East and South-Muntenia. Regarding the prices, the ANOVA table mentioned that the Central region registered the lowest price of medicinal oxygen, while Bucharest-Ilfov and North-East were considered to be the regions with the highest values for unitary price (Table 2). 

When asked if they have been using medicinal oxygen of purity 93% ± 3%, almost 34% of the Romanian hospitals declared to use it during their treatments, while significant differences among regions were revealed by the statistically significant value of the Kruskal–Wallis test (Chi-Square = 13.58, *p*-value = 0.059) at the significance level of 10%. Therefore, the highest prevalence of medicinal oxygen of purity 93% ± 3% was recorded in North-West and Central regions, while at the opposite side, there were North-East and West regions. Also, discrepancies have been signaled regarding the utility of this type of product as the same therapeutic purpose as 99.95% purity oxygen produced industrially. Hospitals from South-East, North-West and Center were more likely to agree with this statement. 

### 4.3. The Segmentation of the Medicinal Oxygen Market Based on Hospital Capacity, Competence Class, Hospital Specialization and Suppling Method 

Analyzing the differences in annual consumption of medicinal oxygen per number of beds, and respectively the unitary price, statistically significant differences can be pointed out regarding the annual consumption and the price of medicinal oxygen depending on the competence class of the hospital. Thus, as expected, the hospitals with a very high and high competence also have the highest level of consumption per bed and also the highest price compared with the hospitals with basic or limited competence class. The value of Pearson’s correlation coefficient (−0.44) between the annual consumption reported to the number of beds and the type of competence of the hospital revealed the existence of a positive relationship; therefore, a higher level of hospital competence meant a higher level of annual consumption reported for the total number of beds.

Also, regarding the main specializations, significant statistical differences could be highlighted, with institute and emergency hospitals being the ones with the highest annual consumption reported to the total number of beds, while at the opposite side, there were specialty and penitentiary hospitals with the lowest consumption per bed. Also, the price led to differences, with institute, emergency and general hospitals registering the highest price, while the specialty and penitentiary hospitals revealed the lowest prices.

**Figure 2:**
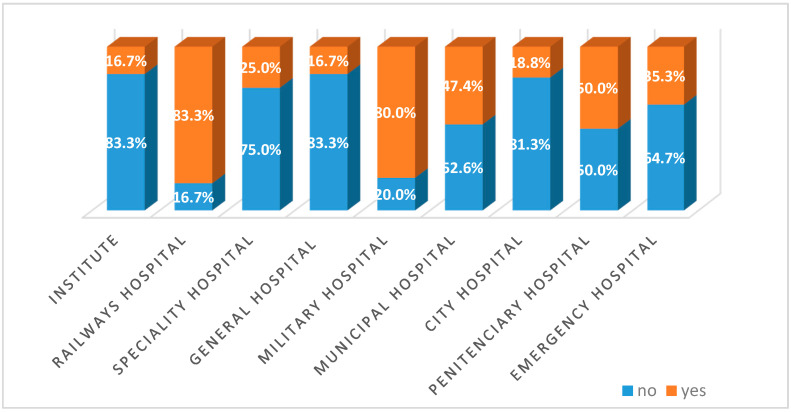
The prevalence of medicinal oxygen of purity 93% ± 3% by specialization.

The majority of hospitals using medicinal oxygen of purity 93% ± 3% have a limited competence, and the empirical results do not support any segmentation based on the competence class. The Kruskal–Wallis test (Chi-Square = 17.71, *p*-value = 0.024) pointed out statistically significant differences regarding the usage of medicinal oxygen of purity 93% ± 3% in different types of hospitals. Therefore, an overwhelming proportion of military and railway hospitals (more than 80% of them) declared to use medicinal oxygen of purity 93% ± 3%, while at the opposite side, there were institutes, general hospitals and city hospitals who declared to use this type of product only in a small proportion (Figure 2).

Also, significant differences were found among different supply methods of medicinal oxygen pointed out by the results of the Kruskal–Wallis test. Thus, most of the hospitals using oxygen of purity 93% ± 3% used the oxygen installations as a method for supply. The main suppliers of medicinal oxygen of purity 93% ± 3% were Microcomputer Service, the main leader in the market for this type of product, GBIndco and Oxistar. When asked if 93% ± 3% purity medicinal oxygen produced on the spot serves the same therapeutic purpose as 99.95% purity oxygen produced industrially, almost 78.5% of the hospitals declared to agree with this.

Thus, hospitals from South-East, North-West and Center were more likely to agree with this statement. Also, regarding the competence class, there were some statistically significant differences revealed by the high significant value of the Kruskal–Wallis test (Chi-Square = 6.95, *p*-value = 0.03). Thus, hospitals with high and limited competence were more likely to consider that these two products could serve the same therapeutic purpose. Regarding the specialization of the hospitals, the empirical results did not support any differences regarding the utility of these two products.

Testing their knowledge regarding the fact that the 93% ± 3% purity medicinal oxygen is considered to be a medicine, being introduced in the Monograph of the European Pharmacopoeia from 2010, almost 60% of the managers declared to know this and to agree with this statement. This statement is largely agreed with by hospitals with high and limited competence, with statistical differences being highlighted by the high value of the Kruskal–Wallis test (Chi-Square = 4.42, *p*-value = 0.045).

Statistical differences have also been captured regarding the socialization of the hospitals. Managers from penitentiary, municipal, railway and military hospitals were more inclined to agree with this statement, while at the opposite side, there were general, specialty and emergency hospitals.

### 4.4. The Segmentation of the Medicinal Oxyhen Market by the Main Suppliers

The main suppliers acting in the Romanian medicinal oxygen market are:

- Multinational companies producing oxygen by the industrial method (99.5% purity): Linde Gaz Romania, Messer Romania, Siad Romania, Air Liquide Romania.
- A Romanian company that uses oxygen generators (purity 93% ± 3%): Microcomputer Service SA.

**Figure 3:**
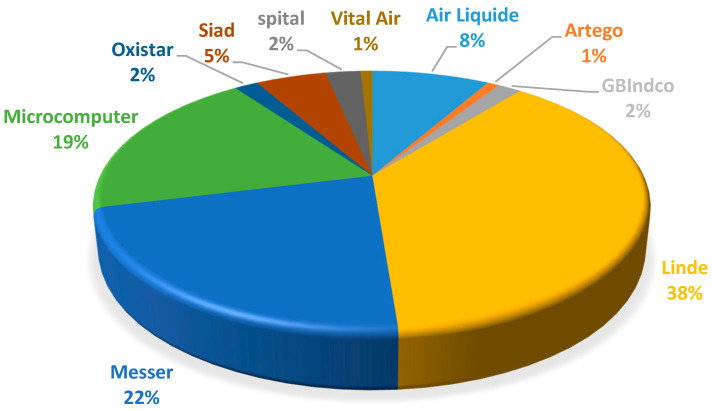
Main suppliers in the Romanian market of medicinal oxygen.

In the last 6 years, 2 companies have appeared interested in entering the medicinal oxygen market, GB Indco SRL and Oxistar SRL. GB Indco SRL from Bucharest initially started by selling medicinal oxygen production facilities, and Oxistar SRL from Cluj mainly serves its own county, having approximately 15 customers. Therefore, the market was mainly divided between Linde and Messer, producing oxygen by the industrial method (99.5% purity), and Microcomputer Service using oxygen generators (purity 93% ± 3%) (Figure 3).

Analyzing the segmentation of the oxygen market based on the main suppliers and the competence class of Romanian hospitals, it can highlighted that Linde Gaz, the main leader in the market, supplies 75% of the health units with very high competence and 53.84% of the hospitals with medium competence, while Messer, the second player in the market, mostly supplied the hospitals with limited competence.

Of the 23 hospitals supplied by Microcomputer, it is present in a percentage of 47.82% in health units with basic competence class and of 26.08% in health units with high competence class.

The segmentation of the medicinal oxygen market based on the main suppliers and the specialization of Romanian hospitals revealed that Linde Romania supplies medicinal oxygen mainly to emergency hospitals (21.7%), while Messer and Microcomputer in high proportions to specialty hospitals (63% and 34.8%, respectively).

The main suppliers of the particular type of product—the medicinal oxygen of purity 93% ± 3%, were Microcomputer Service, the main leader on the market for this type of product, GBIndco and Oxistar.

### 4.5. What Are the Main Differences in the Supplying Method Based on Hospital Comptence Class and Specialization?

Regarding the supply of medicinal oxygen, there was a balance distribution among different ways of supplying the medicinal oxygen on the Romanian market. Thus, 27% of hospitals used concentrators, while 26% of them used oxygen installation, 24% of them have been supplied through storage and only 23% through tubes.

Most of the hospitals with medium (77%) and high (68%) competence used concentrators as the main supply method, while hospitals with a rather limited competence used storage and tubes as supply methods for medicinal oxygen. The highly significant value of the Kruskal–Wallis test (Chi-Square = 80.08, *p*-value = 0.00) revealed statistically significant differences among hospitals from distinct classes of competence regarding the supply method. If institute hospitals as well as general and emergency hospitals use mostly concentrators, storage is used by the city and penitentiary hospitals. The health units that are supplied through oxygen generators are municipal, military, penitentiary and railway (CF) hospitals, these being mostly included in category IV, with basic competence. The sanitary units supplied with oxygen through the tubes are the specialty and city hospitals. Also, in this case, the highly significant value of the Kruskal–Wallis test (Chi-Square = 65.85, *p*-value = 0.00) revealed statistically significant differences among hospitals with different specialization regarding the supply method. 

### 4.6. Identifying the Drivers of the O93% Medicinal Oxygen Market

Putting together all the valuable information regarding the utility and usage of the medicinal oxygen of purity 93% ± 3%, we can mentinon the following:

- Almost one third of Romanian hospitals declared to use it during their treatments, with high prevalence in North-West and Central regions, in hospitals with limited competence and in military and railways hospitals.
- Most of the hospitals using oxygen of purity 93% ± 3% used the oxygen installations as a method for supply.
- The main suppliers of medicinal oxygen of purity 93% ± 3% were Microcomputer Service, the main leader on the market for this type of product, GBIndco and Oxistar.
- Most often, hospitals with high and limited competence as well as penitentiary, municipal, railways and military hospitals were more inclined to agree with the fact that the 93% ± 3% purity medicinal oxygen is considered to be a medicine, being introduced in the Monograph of the European Pharmacopoeia from 2010.
- High and limited competence hospitals’ managers agree that 93% ± 3% purity medicinal oxygen produced on the spot serves the same therapeutic purpose as 99.95% purity oxygen produced industrially.

In this context, it becomes relevant to explore the main factors related to the usage of O93% medical oxygen in Romanian hospitals, aiming to investigate how the regional dimension could impact the usage of this type of product, thus offering valuable information for other potential companies who want to enter the market. 

The empirical results of a null baseline random intercept model revealed the adequacy of such a model, while the log-odds of using O93% medical oxygen in an “average” region was estimated to be β_0_ = −0.71.

The between-region variance of the log-odds of using O93% medical oxygen was estimated as 0.262, with a standard error of 0.31. The significance of the between-region variance has been tested using a Wald test, with the empirical results (Chi-square test = 1.77 with a *p*-value = 0.09) revealing that there is a significant variation between Romanian regions in the prevalence of using O93% medical oxygen.

Based on the value of between-region variance (0.262), the variance partition coefficient (VPC) was computed to be 7.37%, thus 7.37% of the residual variation in the decision of using O93% medical oxygen is attributable to unobserved region characteristics, indicating that almost 8% of the variance in using this type of product can be attributed to differences between regions. 

The empirical results revealed that the main drivers in explaining the usage of O93 oxygen are as follows, and presented in model I (Table 1):

- Considering O93 medical oxygen as a medicine improved the potential usage of this product in treating patients.
- Unitary price has an important role in this equation, exhibiting an expected inverse and statistically significant impact on the decision of using this type of product.
- This usage of this type of product is more prevalent in railway and military hospitals.
- The hospital capacity influenced the prevalence of this product: an increase in the number of operating rooms will definitively increase the probability of adopting this type of product.

The empirical results do not support the impact of hospital competence class, number of beds, the supply method, changes in medical oxygen consumption or a future expansion of the hospital due to the coefficients’ lack of statistical significance.

In this study, we have proven that the decision of using O93 medical oxygen depends on different hospital characteristics, and this was achieved by allowing the models’ intercept to vary randomly across regions in random intercept models. We assumed that the effects of hospital characteristics are the same in each region, i.e., the coefficients of all explanatory variables are fixed across regions. 

Next, we will extend the model by allowing both the intercept and the coefficient of unitary price as well as hospital capacity to vary randomly across regions, making the assumption that the probability of using this oxygen could vary from region to region depending on the variations in prices and hospital capacity, with two random slope models being estimated as models II–III in Table 1. The empirical results of the LR (Likelihood-ratio) test highlighted that the effect of price and hospital capacity varies across regions.

For region j, the effect of a one-unit increase in price on the log-odds of using O93 medical oxygen is estimated as to be −4.80 + $u_{1j}$. The intercept variance of 3.14 is interpreted as the between-region variance in the log-odds of using O93 at the mean price of hospital, while the slope variance of 1.24 is the between-region variance in the effect of price.

The negative intercept-slope covariance estimate implies that regions with above-average probability of using O93 (intercept residual $u_{0j}$ > 0) also tend to have below-average effects of unitary price (slope residual $u_{1j}$ < 0).

For region j, the effect of a one-unit increase in hospital capacity on the log-odds of using O93 medical oxygen is estimated as to be 0.21 + $u_{1j}$. The intercept variance of 1.11 is interpreted as the between-region variance in the log-odds of using O93 at the mean capacity of hospital, while the slope variance of 1.69 is the between-region variance in the effect of total number of operating rooms.

The positive covariance between the intercepts and slopes implies that regions with a high intercept (higher than average probability of using O93, i.e., u_0j_ > 0) also tend to have a steep slope (strong positive relationship between using O93 and the number of operating rooms, i.e., u_1j_ > 0). 

Based on the LR test, testing the null hypothesis of no region variation in the difference between different categories of prices and hospital capacity, we can conclude that the decision of using O93% medical oxygen varies across regions due to variations in price and hospital capacity.

### 4.7. Perspectives of the Romanian Medicinal Oxygen Market

The Romanian market of medical oxygen maintains the growth trend registered globally, with development perspectives for competitors. About half of the Romanian hospitals registered increases in the oxygen consumption and approximately 86% of the hospitals consider that the oxygen consumption will increase in the future by about 5%, this is available mostly for specialized and municipal hospitals. This growth trend is normal, as specialized and municipal hospitals have expanded their scope of intervention, modernized and developed their wards and operating rooms, set up new departments and aimed to increase the degree of competence. Depending on the accreditation process performed, the hospitals were empowered regarding the quality system of the medical health services corresponding to the classification of the units, with this certification giving them the right to conclude contracts with the county health insurance houses. Larger increases of 5% to 10% are stipulated only by the emergency hospitals.

When asked about a future expansion of the hospital, 83.3% of the hospitals that estimate an expansion process estimate that they will register an increase in the annual consumption of medical oxygen between 1% and 5%, while only 16.7% of the hospitals considered that the increase will be at most 10%.

Also, most of institute, railway and emergency hospitals estimate a future expansion in the period 2018/2020, and from these, emergency and institute hospitals estimate an increase in the future consumption between 1% and 5%.

From those hospitals registering increases in the medical oxygen consumption in the first quarter of 2017 compared to the fourth quarter of 2016, almost 51% of them declared to have used 93% ± 3% purity medical oxygen, while almost 61% of those hospitals using this type of product agreed with the fact that their increases in the consumption would be around 5% at most.

Another interesting result was related to the fact the majority of hospitals (57%) estimating an increase in the consumption due to the future expansion of the hospital between 1% and 5% acknowledged that they have been using medical oxygen of 93% ± 3% purity.

## 5. Discussion

This research relied on a quantitative survey conducted based on a structured questionnaire among 121 sanitary units from a statistical population of 461 public hospitals. The questionnaire based on 12 items was sent by email, with the questions being formulated precisely, without ambiguity, so as to ensure that the necessary data were obtained to meet the research objectives.

The sampling was of probabilistic stage-type stratified. The sampling layers targeted were hospital county distribution, hospital competence class and area of residence (urban/rural). The data collection was undertaken during the period March–July 2018. 

Summarizing the most relevant empirical results of our research, we can mention the following. 

There is a regional segmentation of this particular market, relevant from the perspective of identifying the main regional or county poles that could attract the interest of suppliers regarding the hospital profitability in the area, in terms of medical oxygen acquisition. 

Therefore, Bucharest-Ilfov and North-East were the regions with the highest level of average consumption reported to the total number of beds, while the Central region registered the lowest price of medicinal oxygen and Bucharest-Ilfov and North-East the highest values for unitary price. The results subscribe to Garattini’s study conclusions, according to which oxygen consumption has particularities depending on the area [37].

For the usage of this particular product (O93% medicinal oxygen), the regional component also presents relevance, with the highest prevalence of medicinal oxygen of purity 93% ± 3% and also the utility of this type of product as the same therapeutic purpose being recorded in North-West and Central regions.

Therefore, the hypothesis H1 was supported by the results of the Kruskal–Wallis test.

There is a segmentation of the medicinal oxygen market based on hospital capacity, competence class, hospital specialization and supply method. Hospitals with a very high and high competence also have the highest level of consumption per bed and the highest price but are also the ones considering both types of medicinal oxygen as serving the same therapeutic purpose and considering the 93% ± 3% purity medicinal oxygen to be a medicine. The same results were found by Dobson [69]. Also, the price led to differences, with institute, emergency and general hospitals registering the highest price, while the specialty and penitentiary hospitals registered the lowest prices.

By specialization, institute and emergency hospitals have the highest annual consumption reported to the total number of beds, and also the highest price. The prevalence of O93 medicinal oxygen is widespread in military and railway hospitals. 

Therefore, the hypothesis H2 was supported by the results of the Kruskal–Wallis test.

Main suppliers divide the oxygen market using different supply methods and supplying hospitals with different specializations. Hypothesis H3 was supported by the highest proportion of responses, with Linde Gaz being the main leader on the market and supplying mostly very high competence hospitals and almost half of the hospitals with medium competence, and Messer is the second player on the market, mostly supplying the hospitals with limited competence.

While Linde supplies medicinal oxygen mainly to emergency hospitals, Messer and Microcomputer worked with specialty hospitals. 

Regarding the particular type of medicinal oxygen, O93, the main suppliers were Microcomputer Service, GBIndco and Oxistar, and Microcomputer Service SA has the largest market share in supplying oxygen to hospitals through generators.

There is a market segmentation based on supply method, hospital competence class and hospital specialization. Therefore, while medium- and high-competence hospitals, institute and emergency hospitals usually used concentrators as the main supply method, hospitals with a rather limited competence, city and penitentiary hospitals used storage and tubes as the supply method for medicinal oxygen. Oxygen generators are mostly used by municipal, military, penitentiary and railway (CF) hospitals, and oxygen tubes by specialty and city hospitals. The hypothesis H4 was supported by the empirical results of the Kruskal–Wallis test.

The main drivers influencing the decision of adopting O93% medicinal oxygen refers primarily to price and hospital capacity, followed by the hospital specialization, the perception that this product is labelled as a medicine and also to the future perspectives of the medicinal oxygen market. The hypothesis H5 was supported by the statistically significant coefficients of multilevel models, both random intercept and random slope models, supporting the existence of regional disparities regarding the prevalence of using O93% medicinal oxygen among Romanian hospitals, different from Garattini’s study [37].

### Limitations of the Study

All healthcare units in Romania use the national electronic public procurement platform in order to purchase medicinal oxygen from approved drug suppliers. In PEPS (Public Electronic Procurement System), however, there are no statistics on the annual value of contracts concluded between hospitals and suppliers, the quantities of oxygen consumption estimated by hospitals or the number of suppliers. In fact, this information is not available even in the database of the National Institute of Statistics, the main producer of official statistics, who should collect this information and make it available to all categories of users, for planning and research purposes.

The only information available is the number and size of hospitals, which are published on the website of the Ministry of Health, in the form of lists, in which public hospitals are classified according to region, degree of competence and county.

## 6. Conclusions

Most living things need oxygen to survive, and oxygen’s importance in the field of healthcare cannot be underestimated. Oxygen is widely used in every healthcare setting, with applications from resuscitation to inhalation therapy. Oxygen is a vital gas that is of great significance in medical care throughout the world, from routine use and home medical care to more urgent treatment. According to studies, survival in patients with hypoxia (COPD or sleep apnea) can be prolonged by several years if the patient receives regular oxygen therapy. 

Medicinal oxygen is the primary treatment administrated to the majority of patients suffering from respiratory problems and low levels of oxygen in the blood, and in the context of the actual health crisis caused by the COVID-19 pandemic, a challenge is represented by increasing the supply of medicinal oxygen while reducing cost so that it is accessible where it is needed most, free at the point of use. It will take increased investment and commitment to put oxygen at the center of strategies for universal health coverage. In this context, it becomes essential to investigate the main characteristics of the Romanian market of medicinal oxygen, highlighting top key players, market development, key driving factors, types of products, market perspectives as well as shedding light on the segmentation of this particular market based on considerations regarding regions, hospital competence class and hospital specialization. Also, this research aimed to explore the regional disparities in the decision of using O93% medicinal oxygen, revealing the main factors related to the usage of this type of product among Romanian public hospitals.

The empirical results revealed that currently, two types of medicinal oxygen are used in the hospital system in Romania: medicinal oxygen O99.5%, produced according to liquefaction technology, being obtained industrially and delivered in tubes and tanks, and medicinal oxygen O93%, being produced according to the technology of molecular sieves and using medicinal oxygen concentrating devices, which allows the preparation of medicinal oxygen right inside the hospitals.

The two products are used for the same medical purpose, being substitutable, the first being a drug in the European Pharmacopoeia, the second being a drug in the American Pharmacopoeia since 1990, produced and marketed in Romanian hospitals since 2000, in accordance with Law 176/2000, Directive European Association of Medical Devices 93/42/EEC and the international medical standard SR EN ISO 7396-1: 2006.

This research revealed the existence of medicinal oxygen market segmentation based mostly on hospital competence class and specialization, hospital capacity, supply method or regional dimension.

Firstly, this information is valuable for potential suppliers that aim to enter this market, in terms of medical oxygen acquisition. In such a context, it is very important to know that Bucharest-Ilfov and North-East are the regions with the highest level of average consumption reported to the total number of beds, but also with the highest price. This particular type pf product (O93% medicinal oxygen) is more prevalent in in North-West and Central regions, being used for therapeutic purposes.

High-competence hospitals as well as institute and emergency hospitals have the highest amount of consumption per bed, but also the highest price. However, O93% medicinal oxygen is more often used in military and railways hospitals. 

The market is divided into a small number of suppliers. While Linde Gaz is the main leader in the market supplying mostly high-competence hospitals and emergency hospitals, Messer and Microcomputer worked with specialty hospitals with limited competence, with Microcomputer Service SA having the largest market share in supplying oxygen to hospitals through generators.

Also, the supply method created a split of the market. While high-competence hospitals as well as institute and emergency hospitals usually used concentrators as the main supply method, limited competence hospitals as well as city and penitentiary hospitals used storage and tubes as the supply method for medicinal oxygen.

The regional discrepancies in the usage of O93% medicinal oxygen have been explained firstly by the price and hospital capacity and secondly by the hospital specialization, the medicine label of O93% oxygen as well as by the future perspectives of the medicinal oxygen market. 

The results obtained from the analysis are important in the healthcare management, because the health unit, having the quality of a public institution financed from the state budget, should identify cost-effective solutions and generate savings in conditions of equivalent quality. Thus, following the information, one can have access to market opportunities or constraints, information about the participating suppliers, the prices charged according to the oxygen supply methods and the specificities of the environment in which the participants act.

Information on the monthly oxygen consumption of hospitals, the monthly value spent on supply, the ranking of this data and the interpretations of the data collected can also lead to an improvement in the public procurement process.

The activity of control structures in the field of public procurement does not focus enough on the quality–price ratio that underlies the procedures, the principles of transparency and an adequate competitive framework, with this being oriented mostly on non-essential issues and exaggerated in importance, where respecting it is the letter of the law rather than its meaning. Public procurement is an essential aspect of public investment, it stimulates economic development and is an important element for boosting the oxygen market.

Public health units purchase goods and services, but this must be done in the most efficient way. The effect obtained is to establish a competitive environment in order to develop, obtain and maintain optimal results in the market and to eliminate and restrict activities that discriminate against potential bidders.

Given that the price of oxygen produced by generators is below the price of industrial oxygen, being financially advantageous at least for a certain category of hospitals, all health units should be interested in this product, aiming at the principle of efficient use of public funds, since it is known that they are always insufficient in relation to the needs and supervision of the costs of the procurement procedure.

Based on the information obtained, the health units balance their budgets and establish development policies in order to optimize health services, so that, at the central level of the ministry, hospital management plans are integrated into government programs dedicated to health policies, with objectives strategies and action plans.

## Figures and Tables

**Table 1:** Multilevel logistic regression on O93% medicinal oxygen.

Variable	Model I		Model II		Model III	
	β	exp ^1^	β	exp	β	exp
O93% medicinal oxygen is a medicine (no) Yes Price (M^3^)	3.44 *** −4.14 ***	31.26 0.02	3.81 *** −4.8 ***	45.36 0.001	3.65 *** −4.4 ***	38.62 0.01
Number of operating rooms	0.19 ***	1.21	0.23 ***	1.26	0.21 ***	1.23
Railway hospital (otherwise) Railway	5.97 ***	391.72	7.12 ***	1232.72	6.45 ***	634
Military hospital (otherwise) Military hospital	5.55 ***	256.09	7.02 ***	1117.8	6.38 ***	587.92
Constant	3.93 ***	50.86	4.68 ***	107.95	4.09 ***	59.36
Observations	121		121		121	
No. of groups	8		8		8	
Log likelihood	−29.36		−28.76		−28.97	
Wald chi ^2^	25.27		15.87		20.88	
	Random part identity: Regions					
Variance (constant)	0.04		3.15		0.01	
(intercept variance) (Standard Error)	0.6		7.26		0.15	
Variance at regional level ^2^ (%)	1.2%		48.91%		0.3%	
LR test	0.01		1.2		0.8	

*** *p* < 0.01, ^1^ Odds ratio. ^2^ Variance partition coefficient: measures the proportion of the total residual variance that is due to between-group variation. Note: All coefficients are compared to the benchmark category, shown in brackets. All country level indicators were centered to the mean obtained using a weighting scheme. LR represents Likelihood-ratio.

**Table 2:** The empirical results of the analysis of variance (ANOVA) for regional discrepancies in consumption and price of medicinal oxygen.

Regional Discrepancies on Consumption and Price of Medicinal Oxygen						
		Sum of Squares	df	Mean Square	F	Sig.
Annual consumption of medicinal oxygen/number of beds	Between Groups	344,543.872	7	49,220.553	1.884	0.079
	Within Groups	2,952,795.876	113	26,130.937		
	Total	3,297,339.748	120			
Unitary price (RON/m^3^)	Between Groups	9.330	7	1.333	2.902	0.008
	Within Groups	51.901	113	0.459		
	Total	61.232	120			
The Distribution of Consumption and Price of Medicinal Oxygen by Competence Class of Hospitals						
Annual consumption of medicinal oxygen/number of beds	Between Groups	778,175.227	4	194,543.807	8.958	0.000
	Within Groups	2,519,164.521	116	21,716.936		
	Total	3,297,339.748	120			
Unitary price (RON/m^3^)	Between Groups	8.246	4	2.062	4.513	0.002
	Within Groups	52.985	116	0.457		
	Total	61.232	120			
The Distribution of Consumption and Price of Medicinal Oxygen by Hospital Specialization						
Annual consumption of medicinal oxygen/number of beds	Between Groups	1,162,411.035	8	145,301.379	7.623	0.000
	Within Groups	2,134,928.713	112	19,061.864		
	Total	3,297,339.748	120			

**Table 3:** Descriptive statistics for annual consumption of medicinal oxygen and unitary price.

		Annual Consumption of Medicinal Oxygen/Number of Beds	Unitary Price (m^3^)
Mean		146.029	2.159
Median		96.000	2.121
Mode		120.00	2.0
Minimum		8.09	0.8
Maximum		1425.00	4.0
Percentiles	25	57.175	1.628
	50	96.000	2.121
	75	205.535	2.749

## Supplementary Materials

The following are available online at https://www.mdpi.com/2227-9032/9/2/155/s1.

## Appendix A

**Table A1:** Variables description.

Variables	Variable Type
average monthly and annual medicinal oxygen consumption	numerical variables
average monthly and annual amount spent by the hospital for the supply of medicinal oxygen in the previous year	numerical variables
unitary price of a m^3^	numerical variables
the way the hospital was supplied with medicinal oxygen	categorical variable: value 1 for oxygen tank, value 2 for oxygen installation, value 3 for storage, installation, value 4 for oxygen tanks and value 5 for medicinal oxygen concentrators
the incidence of 93 ± 3% Purity Medicinal oxygen in the Romanian hospitals	dichotomous variable: value 1 for yes and value 0 for no
the utility of using 93 ± 3% Purity Medicinal oxygen	rated on a five-point Likert scale, with value 1 for totally agree and value 5 for totally disagree
93 ± 3% vs. 99.95% Purity Medicinal oxygen	dichotomous variable with value 1 for yes and value 0 for no.
the importance of the medicinal oxygen	dichotomous variable with value 1 for yes and value 0 for no
the changes in the medicinal oxygen consumption	categorical variable with value 1 for it increased, value 2 for it decreased, value 3 –it remains unchanged
the relative change in the medicinal oxygen consumption	categorical variable with value 1 for 1–5%, value 2 for 5–10% and value 3 for more than 10%
the potential future hospital expansion	dichotomous variable with value 1 for yes and value 0 for no
the amount of the annual increase in the medicinal oxygen consumption	categorical variable with value 1 for 1–5%, value 2 for 5–10% and value 3 for more than 10%
the current medicinal oxygen supplier	categorical variable with value 1 for Messer, value 2 for Linde, value 3 for Microcomputer, value 4 for SIAD, value 5 for GBIndco, value 6 for Oxistar, value 7 for Air Liquid, value 8 for hospital, value 9 for Vital Air and value 10 for Artego
hospital specialization	categorical variable with value 1 for institute, value 2 for clinical hospital, value 3 for specialty hospital, value 4 for general hospital, value 5 for military hospital, value 6 for municipal hospital, value 7 for city hospital, value 8 for penitentiary hospital and value 9 for emergency hospital
competence hospital class	five –point Likert scale with value 1 for very high competence and value 5 for limited competence
number of beds	numerical variable
number of operating rooms	numerical variable
