# Supplementary material for: Shedding Light on the Main Characteristics and Perspectives of Romanian Medicinal Oxygen Market
Source: Healthcare (Basel). 2021 Feb 3;9(2):155. doi: 10.3390/healthcare9020155 (PMC7913192; doi:10.3390/healthcare9020155)
Supplement: Supplementary file 1 [file healthcare-09-00155-s001.pdf]

Supplementary Material

1. The first medical decision at European level on equal treatment between O93 and O99.5

Medicinal oxygen O93 is listed as a drug in the American Pharmacopoeia, the Compendium of Drug Information for the United States, which sets standards for medical products, supplements and ingredients, medical devices for which the FDA (The Food and Drug Administration) is responsible.

The legal working document of the European Directorate for the Quality of Medicines and Healthcare (EDQM, body of the Council of Europe) was the basis for the introduction of the O93% monograph in the European Pharmacopoeia in March 2010 in Strasbourg. The pharmacopoeia is the scientific and legal standard for quality control during the processes of development, manufacture and marketing of medicines. All manufacturers of medicines are obliged to apply this quality standard in order to distribute the products in the countries of the European Union.

Considering that the monographs of the pharmacopoeia represent an official standard, the regulation regarding the quality of oxygen medicine was implemented directly in the national legislation by the Health Law 95/2006, republished regarding the health reform.

In the process of transferring the oxygen monograph from the American Pharmacopoeia to the European Pharmacopoeia, the results and conclusions of the Microcomputer Service Company that used in Romania the oxygen produced by generators, since 2000, were also presented.

Each type of oxygen corresponds to a monograph in the Pharmacopoeia, as follows: the medicinal oxygen produced by O93 generators is found in monograph 2455, and the liquefied medicinal oxygen, industrial product in monograph 0417.

Both monographs state that the two types of oxygen are intended for medicinal use, which implies that they are similar products and replace the same purpose. Although the medicinal quality of medicinal oxygen O93 was clearly legislated by the only reference work for the control of drug quality in the European Union, it became mandatory for all signatory states of the convention, however, the National Agency for Medicines and Medical Devices in Romania argued that the two types of oxygen are qualitatively different, that O93 oxygen is not a drug and assumptions are made of an alleged legal and therapeutic similarity with liquefied oxygen.

Since the introduction of the O93 medicinal oxygen monograph in the European Pharmacopoeia, the health units have gradually introduced in the public procurement documentation the need to comply with the compliance of oxygen with the provisions of the Pharmacopoeia.

2. The first legal decision at European level on equal treatment between O93 and O99.5

The oxygen market disproportionate to the influences of large companies trying to remove any new supply of medicinal oxygen, had to be balanced taking into account the offers of other participants. Barriers to entry on this market were also exerted by pressure from multinationals on state bodies such as NAFA (National Agency of Fiscal Administration), the Ministry of Health, the Financial Guard, the Ministry of Interior, through complaints about the quality of oxygen O93.

The Association of Industrial Gas Producers in Romania and Linde Gaz Romania complained to the Ministry of Public Finance, General Directorate of Public Finance of Bucharest that medicinal oxygen O93 cannot be classified as medicinal oxygen, cannot be authorized as medicine, is not regulated for use medicinal product in the European Pharmacopoeia and requested investigations into the activity of Microcomputer Service.

In order to counteract these manifestations of limitation of the right to use medicinal oxygen O93 and to prove that the accusations of the competitors are unfounded, a long-term lawsuit (5 years) was reached between Microcomputer Service and NAFA - Regional General Directorate of Craiova Public Finance.

Romania is the only country in the European Union where such a process took place, the company having the merit of being the first making efforts to regulate the quality of O93 oxygen medicine, so that it can be used in health facilities , in all medical procedures, as a therapeutic product equivalent to industrial oxygen. The lawsuit was aimed at demonstrating equal treatment between the medicinal oxygen produced by generators and liquefied industrial oxygen, legislating the quality of O93 oxygen medicine and annulling the tax control act, which regulated the financial regime related to the payment of VAT. Given that in Romania different VAT rates are applied depending on the CANE codes (fields of activity), the medicinal quality of medicinal oxygen O93 also implies a VAT rate of 9%, compared to the percentage of 19% VAT, in the situation in which the product is not considered a medicine.

The inclusion of O93 oxygen in the category of medicines or the exclusion from this category would have had major legal consequences on the level of taxation of the company.

Thus, following the complaints sent by the multinational companies and the Association of Industrial Gas Producers in Romania to the local financial departments, to the National Agency of Medicines and Medical Devices, and to the health units in the country, the Ministry of Public Finance notified itself and initiated the verification to fulfill the fiscal obligations provided by the legislation regarding the collection and recovery of the VAT rate of the Microcomputer company. Given the attractiveness of the oxygen market and the monopoly exercised in this market around 2000 by two large multinational industrial oxygen production companies, their interest was to prevent any other competitor from entering the market or to share it with other interested companies. An advantage of the multinationals was at that time the fact that the oxygen produced by the generators was not yet included in the European Pharmacopoeia and its quality was not very well regulated in Romania.

Thus, during the five years of trials, Microcomputer Service showed that in the Romanian medical system two types of oxygen are used for medicinal use, these being two similar products, which are in direct competition on the market and must enjoy equal treatment. He argued that O93 oxygen is a medical product obtained by modern methods, by medical devices, implicitly at much lower costs,

The company also presented to the court medical technical expertise performed by a specialist in chemistry and engineering of organic substances and extensive evidence that contained scientific papers on oxygen O93. The objectives of the expertise were, among others, if O93% oxygen has effects similar to those of O99%, if O93% does not require a marketing authorization and if O93 corresponds to the definition of the drug, by strict reference to the provisions of art. 695 of Law 95/2006.

Another argument arose from the application of the European Directive 2006/112 / EC on the common system of value added tax, which requires neutral and uniform taxation of like products in the European Union and in the territory of the Member States.

In fact, the Ministry of Health also agreed to the use of medicinal oxygen O93% + - 3% in hospital units through an address in 2008, stating that the use of medicinal oxygen produced by oxygen concentrators does not require authorization placing on the market by the National Medicines Agency, only the medical device certificate being required.

The case of the company Microcomputer Service finally reached the role of the High Court of Cassation and Justice (HCCJ) which admitted the company's appeal and sent it back to the Court of Appeal for retrial. The HCCJ considered that the argument put forward by Microcomputer, which referred to the case-law of the Court of Justice of the European Union, on the principle of fiscal neutrality, the fundamental principle of the common system of VAT, which opposes, in particular, like similar goods, was relevant and correct. Therefore in competition with each other, to be treated differently from the point of view of VAT.

The Microcomputer Service Company won the lawsuit against the Ministry of Finance by the final sentence, in which the following were established:

-medical oxygen 93% has effects similar to those of 99.5% oxygen because the active substance is the same in both products

- 93% medicinal oxygen does not require a marketing authorization as the authorization of the medical device, ie the oxygen concentrator, is sufficient, the oxygen being obtained through a non-industrial process, and therefore does not require a marketing authorization.

-93% oxygen is a drug according to the definition of the drug art. 695 of Law no. 95/2006

Regulation of the medical oxygen regime 93% + / - 3% was an essential step in the future development of the market for medicinal oxygen produced by concentrators, especially given the economic and financial power of direct competitors. There is an aggression of multinational companies that influences the business environment, although this behavior is counterproductive and allows the spread of a wrong message to society. Normally, there are expectations from multinationals, foreign companies to enter domestic markets with capital, know-how, innovative technologies, and ethical behavior, gaining competitive advantage through superior performance and not through dishonest business practices.

In fact, the activity of foreign companies in Romania has become quite contested, in recent years, in the sense that their presence affects the activity of companies with domestic capital, without the positive effects of the presence of foreign capital in Romania to be significant. The National Bank of Romania itself wrote in the Annual Report for 2018 that "for the Romanian economy was identified an indirect impact (of the presence of foreign companies - no) cumulative almost zero: positive training effects in the relationship between domestic supplier - multinational customer and negative effects on horizontal, between competitors".

Although local authorities and health agencies should normally establish policies, develop national programs to promote partnerships between foreign and local companies or provide assistance to domestic producers, they have supported the interest of multinationals, not being interested in the existence and promotion of a fair competition.

Moreover, a first legislative obstruction was created by the exponent of a state medical institution, the National Agency for Medicines and Medical Devices (NAMMD), which should promote the new on the Romanian medical market, issuing official addresses claiming that oxygen O93 it is not a medicine. He appropriated responsibilities for which he had no competence, transmitted speculations and misinterpretations, indirectly supporting the product of competing companies.

Following the legal approach and the results obtained, the abuse of the dominant position of the large industrial oxygen producing companies was partially eliminated, progressing to equalize the treatment conditions between the two products: medicinal oxygen produced by oxygen generators and liquefied medicinal oxygen industrial product.

Efforts to improve the legislative framework and develop alternatives to oxygen supply hospitals continued beyond the competence of local authorities and the company argued to recognized national and international standardization bodies for clarification on this issue.

3. Personal considerations regarding the improvement of the standard EN 7396-1: 2016 regarding the equal treatment between O93 and O99

The award of the procurement contract is the result of a public procurement process that takes place in several stages, through the Electronic Public Procurement System, especially considering the public quality of most customers of companies in the medical oxygen market. Thus, all public hospitals, as contracting authorities, are obliged to award public procurement contracts using the SEAP platform, having the possibility to choose the procedure as follows:

a) the proposed value of the acquisition;

b) the scope of the contract to be signed with the supplier;

c) Making the provisions for the awarding procedures.

The SEAP platform has the obligation to allow all economic operators registered in the system to access independently and without restrictions the electronic documents of the tenders, ie the data sheet, specifications, DUAE form or other standardized forms.

The legislation specifies the manner of implementation of the procurements, the norms for awarding the contracts, the particular modalities that can be applied for concluding the contracts with the bidders, but also the aspects characteristic of the development of the respective contracts. The law provides the legal framework for the procurement of goods, services and public works. The rules on the basis of

which public procurement agreements are concluded are also defined, of which the principles of non-discrimination and equal treatment are the subject of clarifications and appeals submitted by Microcomputer Service in public procurement procedures.

Contracting authorities are obliged to ensure equal treatment, without discrimination of potential economic tenderers, and to show transparency and proportionality. Hospitals must structure and design procurement so as not to introduce an artificial restriction of competition, in the sense that the procurement or its elements are drafted in such a way as not to unduly favor or disadvantage potential economic bidders. In practice, the company Microcomputer Service frequently encounters the situation of restricted competition, because the company is limited access to that purchase by requiring certain hospitals to purchase only liquefied medical oxygen (99.95%).

The term "acquisition" used in art. 17 paragraph (1) lit. a) of GD no. 395/2016 does not refer to a contract of products, services or works awarded at a certain moment, but represents a concept that relates to / includes products, services and works considered similar. So, in situations where hospitals publish procurement documentation referring in particular to liquefied oxygen, obtained industrially, they exclude from the auction the equivalent product, gaseous medicinal oxygen, 93% + / - 3%, produced by generators, although it is an equivalent therapeutic product, with the same effect in the treatment of patients.

Directive 2014/24 / I defines the concept of 'similar products' as 'those products which cumulatively meet the following conditions:

a) They are intended for identical or similar uses;

b) They are part of the normal range of products that are supplied / traded by economic operators with constant activity in the respective sector.

Medicinal oxygen 93% + / - 3% is provided in the European Pharmacopoeia, Monograph 2455 both as a product and as a technology of obtaining. It has been proven with well-established scientific and medical arguments and legally in Romania, that oxygen 99.5% and oxygen 93% ± 3% are therapeutically equivalent as, moreover, it has been established that oxygen levels 90% or more large, are therapeutically equivalent to 100% oxygen. This system has been used successfully for over 15 years in the Romanian medical system.

The SR EN ISO 7396-1 standard, "medical gas distribution systems", was developed and adopted within a mandate given to CEN (European Committee for Standardization) by the European Commission and the European Free Trade Association and supports the essential requirements of the European Directive 93/42 / EEC on medical devices.

This standard, including the requirements for the design, installation, operation, performance and commissioning of feeding systems used in medical institutions for medicinal oxygen, with national and international reference and recognition status in all medical oxygen auctions, makes serious omissions and confusions on the notion of medicinal gas oxygen 93%.

All errors are due to the fact that this type of medical gas is produced by an oxygen concentrator right inside the hospital units unlike the 99.5% oxygen medical gas that is produced industrially and needs to be transported in cylinders or oxygen tanks in the premises of the hospital units.

Oxygen produced industrially by the cryogenic method has been discovered and used for over 100 years as industrial oxygen, including in hospital units.

The 93% medicinal oxygen produced by concentrators by the PSA method, was discovered and used around 1980, being obtained through a revolutionary technology, in accordance with the scientific and technological evolution of some industrially advanced states.

Like any innovative discovery, the medicinal gas 93% + - 3% oxygen was difficult to assimilate and understand by hospital units and last but not least, it was not approved by industrial oxygen producers, because it is a competing product especially on the market medical.

Perhaps also because of this the SR EN ISO 7396-1: 2016 standard contains ambiguities regarding the definition, utility and use of this new product in hospital units.

Noting these non-conformities, the company Microcomputer Service, having also the quality of ASRO member in the "Medical" technical committee, sent at the beginning of 2020 to the Romanian

National Standardization Body (ASRO) and to the International Organization for Standardization (ISO), proposals for improvement of the standard. These were accepted at national level by ASRO Romania, being subsequently submitted to the analysis of the members of the European Standardization Commission, which represents the European Association of National Standardization Institutes, the National Standardization Bodies. The members participating in the working groups of the CEN Technical Committees are involved in detail in the development of the standards, being obliged to contribute directly as experts and to find the most correct expertise in their own network.

The revision and improvement of the standard is essential due to its importance in ensuring compliance with the principles of transparency and real competition in public procurement procedures, and the efficient use of public funds. Its clarification will lead to the avoidance of confusions and appeals that may arise due to the misunderstanding of the references to the standard or the technical provisions contained.

Thus, the company Microcomputer Service submitted a series of modification proposals, regarding the revision of standard 7396-1: 2016.

The approval of these proposals and the revision of the standard will clarify the notion of medicinal oxygen 93% + / - 3% and will facilitate its introduction in hospital allocation documents, as an equivalent product of liquefied oxygen, serving the same therapeutic purpose, respecting best practices existing in European and international space.
